# Genome architecture changes and major gene variations of *Andrias davidianus* ranavirus (ADRV)

**DOI:** 10.1186/1297-9716-44-101

**Published:** 2013-10-21

**Authors:** Zhongyuan Chen, Jianfang Gui, Xiaochan Gao, Chao Pei, Yijiang Hong, Qiya Zhang

**Affiliations:** 1 State Key Laboratory of Freshwater Ecology and Biotechnology, Institute of Hydrobiology, Chinese Academy of Sciences, Wuhan 430072, China; 2 College of Life Sciences and Food Engineering, Nanchang University, Nanchang 330031, China

## Abstract

Ranaviruses are emerging pathogens that have led to global impact and public concern. As a rarely endangered species and the largest amphibian in the world, the Chinese giant salamander, *Andrias davidianus*, has recently undergone outbreaks of epidemic diseases with high mortality. In this study, we isolated and identified a novel ranavirus from the Chinese giant salamanders that exhibited systemic hemorrhage and swelling syndrome with high death rate in China during May 2011 to August 2012. The isolate, designated *Andrias davidianus* ranavirus (ADRV), not only could induce cytopathic effects in different fish cell lines and yield high viral titers, but also caused severely hemorrhagic lesions and resulted in 100% mortality in experimental infections of salamanders. The complete genome of ADRV was sequenced and compared with other sequenced amphibian ranaviruses. Gene content and phylogenetic analyses revealed that ADRV should belong to an amphibian subgroup in genus *Ranavirus*, and is more closely related to frog ranaviruses than to other salamander ranaviruses. Homologous gene comparisons show that ADRV contains 99%, 97%, 94%, 93% and 85% homologues in RGV, FV3, CMTV, TFV and ATV genomes respectively. In addition, several variable major genes, such as duplicate US22 family-like genes, viral eukaryotic translation initiation factor 2 alpha gene and novel 75L gene with both motifs of nuclear localization signal (NLS) and nuclear export signal (NES), were predicted to contribute to pathogen virulence and host susceptibility. These findings confirm the etiologic role of ADRV in epidemic diseases of Chinese giant salamanders, and broaden our understanding of evolutionary emergence of ranaviruses.

## Introduction

Iridoviruses are associated with numerous mortality events in aquaculture and wildlife conservation [1-3], and have been recognized as killers for economically and ecologically important poikilotherms including fish, amphibians and reptiles [4-6]. Their host ranges and virulence vary widely among the described iridoviruses [4,6,7]. Among five genera in the family *Iridovidae*, the genus *Ranavirus* includes the majority of iridoviruses [8]. And, several mass mortality and amphibian population declines in different worldwide locations were reported to be caused by epidemic widespread and outbreaks of different ranaviruses, such as frog virus 3 (FV3) [9,10], *Ambystoma tigrinum* virus (ATV) [11,12], *Rana grylio* virus (RGV) [13,14], tiger frog virus (TFV) [15] and common midwife toad ranavirus (CMTV) [16]. At present, eleven ranaviruses isolated from fish, amphibians and reptiles have been completely sequenced [17-21]. Comparative analysis of ranavirus genomic sequences revealed that the ranaviruses isolated from amphibians have high degrees of genome sequence colinearity [5,18] and CMTV represents an evolutionary intermediate among the sequenced ranaviruses [20].

The Chinese giant salamander *Andrias davidianus*, is the largest amphibian in the world. Because of its natural population decline and high values for scientific conservation and medicinal use, it has been farmed in many locations throughout China. Unfortunately, an outbreak of epidemic disease with high mortality has occurred on most of the farms since 2010. Ranavirus has been suggested as a pathogen for diseases based on major capsid protein (MCP) gene sequence analysis by PCR [22,23], but its pathogen-related biological characteristics, complete genome sequence and organization, and evolutionary position in iridoviruses have remained unknown. Since ranaviruses are known to infect more than 70 amphibian species and to be emerging pathogens that have led to global impact and public concern [24-27], and Chinese giant salamander is a rare and endangered species, comprehensive studies on the newly emerging ranavirus will be of important significance for increased understanding of its emergence and ecology mechanisms as well as its conservation strategies. For these purposes, we investigated the biological features of a novel ranavirus from diseased Chinese giant salamanders, determined and molecularly characterized its complete genome, analyzed the significant genome changes between the virus and other ranaviruses, and identified several major genes contributing to pathogen virulence and species susceptibility.

## Materials and methods

### Ethics statement

All animal procedures were conducted in accordance with the recommendations in the Regulations for the Administration of Affairs Concerning Experimental Animals of China. The protocol was approved by the Institutional Animal Care and Use Committee of the Institute of Hydrobiology, Chinese Academy of Sciences, and all efforts were made to minimize suffering.

### Sample collection

During May 2011 to August 2012, an outbreak of epidemic disease occurred in Chinese giant salamanders from the natural habitats or farms in Hunan, Jiangxi and Henan Provinces of China. Larval and adult salamanders were affected. Generally, the incidence rate in larvae (more than 40%) was higher than that in adults (about 10%), and the mortality was high up to nearly 100% in the diseased salamanders. Ten diseased Chinese giant salamanders (two larvae and eight adults) were collected from the affected areas, and necropsy examination was performed in the virology laboratory at the Institute of Hydrobiology, Chinese Academy of Sciences. Tissue samples were taken for virological and histopathological studies.

### Virus isolation

Liver, spleen and kidney tissues from diseased Chinese giant salamanders were cut into pieces and homogenized in phosphate buffered saline (PBS) with antibiotics (100 IU penicillin mL^-1^, 100 μg streptomycin mL^-1^). Extracts were stored overnight at -20 °C, thawed, clarified by centrifugation at 2000 × *g* and filtered through a sterile 0.45 μm filter (Millipore, Billerica, MA, USA). The filtered supernatant was used as the original viral isolate for infecting cell lines and stored at -80 °C.

*Epithelioma papulosum* cyprini (EPC), Fathead minnow (FHM), Grass carp fins (GCF), Grass carp ovary (GCO), Bluegill fry (BF-2) and Chinook salmon embryo (CHSE) cell lines maintained in our laboratory were used for virus isolation and sensitivity tests. The above tissue homogenates were inoculated onto confluent monolayers of these cells maintained in TC 199 medium with 5% fetal bovine serum at 20 °C. When advanced cytopathic effects were observed, the cell culture supernatants were harvested and used as virus stocks for virus passages and characterization. Virus titers were measured on the basis of 50% tissue culture infective dose (TCID_50_)/mL as described previously [14,28].

### Biophysical and biochemical property detection

The optimal temperature for virus propagation was assayed by infection of EPC cell monolayers at 15 °C, 20 °C and 25 °C. Heat stability was measured by incubating the virus suspension at 56 °C for 30 min, and then the titer was determined. Stability to pH was tested by incubating the virus at 20 °C in TC 199 medium adjusted to pH 3.0 and pH 10.0. After 1 h of incubation, the samples were titrated. Chloroform and 5-iododeoxyuridine (IUdR) sensitivity were determined as described previously [29].

### Electron microscopy

Infected EPC cells were harvested after appearance of CPE by scraping the cells into the medium, followed by centrifugation at 700 × g for 10 min. The pellets were fixed with 2.5% glutaraldehyde, post-fixed in osmium tetroxide (OsO4), dehydrated and embedded in Epon-812 [30,31]. Ultrathin sections were cut and stained with uranyl acetate and lead citrate, and examined with electron microscopy (JEM-1230, JEOL, Tokyo, Japan).

### Experimental infection

Healthy Chinese giant salamanders (mean weight of about 120 g) were divided into two groups of four salamanders each. The salamanders in one group were each injected intraperitoneally with 0.5 mL of virus stock prepared from EPC cell cultures at a concentration of 1 × 10^6^ TCID_50_mL^-1^, and salamanders in another group were injected with PBS as controls. The challenged salamanders were monitored daily, and the moribund salamanders were harvested for histopathological examination and virus re-isolation.

### Histopathology

Tissue samples (liver, kidney, spleen, and intestine) were fixed in 10% neutral buffered formalin, and then routinely processed and embedded in paraffin wax. Sections (5 μm) were stained with hematoxylin and eosin, and examined by light microscopy. Samples from normal healthy salamanders were run in parallel as negative controls.

### Genomic DNA extraction and sequencing

Virus particles were purified from cell culture-amplified virus stocks as described [32,33]. ADRV genomic DNA was prepared from purified virus particles by phenol-chloroform extraction [34]. Sequencing of ADRV genome was carried out using the Roche/454 GS FLX system (Roche-454 Life Sciences, Branford, CT, USA). Briefly, after the quality of ADRV genomic DNA was assessed by agarose gel electrophoresis, 10 μg samples were broken into fragments of 300–500 bp by nebulization. The whole genomic library was amplified using GS emPCR kits and sequenced with 454 GS FLX instrument according to the manufacturer’s instructions. The consensus sequence of the whole DNA sample was generated by assembly of the 454 sequencing data with GS De Novo Assembler software version 2.6 (Newbler 2.6, Roche-454 Life Sciences). The average reading frame length was about 360 bp with 58-fold genome coverage. The gaps were filled with the primer-walking technique.

### Genome annotation and analysis

Genomic DNA composition, structure, nucleotide and amino acid sequences were analyzed with the DNASTAR program (Lasergene, Madison, WI, USA). The open reading frames (ORFs) were predicted using Gene Finding in Viral Genome program [35] and NCBI ORF Finder [36]. ORFs were identified according to the following criteria [18-20]: (1) they were at least 120 bp, (2) they could be detected by the two annotation methods, and (3) they were not located within larger ORFs. Overlapping ORFs were annotated only if they had homologs to other sequenced iridoviruses. Comparisons of homologous sequence regions of ADRV with other viruses were conducted in GenBank database using BLAST programs. Transmembrane domains (TM) were predicted using TMHMM 2.0 [37]. Secondary structures were predicted using the JPred program [38]. Nuclear localization signal (NLS) and nuclear export signal (NES) were predicted using the PredictProtein server [39] and NetNES 1.1 [40], respectively. Multiple sequence alignment was conducted using ClustalX 1.83, and sequence identities were calculated using the MegAlign program. The genome sequence of ADRV was deposited into GenBank under accession no. KC865735.

Phylogenetic analysis was performed based on the alignment of the concatenated sequences of 26 iridovirus core proteins from ADRV and other completely sequenced iridoviruses. The phylogenetic tree was constructed by MrBayes 3.2 using a mixed amino acid model with 150 000 generations and a sampling frequency of 100 [41]. DNA dot matrix plots were obtained using DNAMAN version 6 (Lynnon Corp., Quebec, Canada). Iridovirus sequences used for analysis were obtained from GenBank, and the accession numbers were collected in Additional file 1.

## Results

### Symptoms of disease and histopathological changes

**Figure 1:**
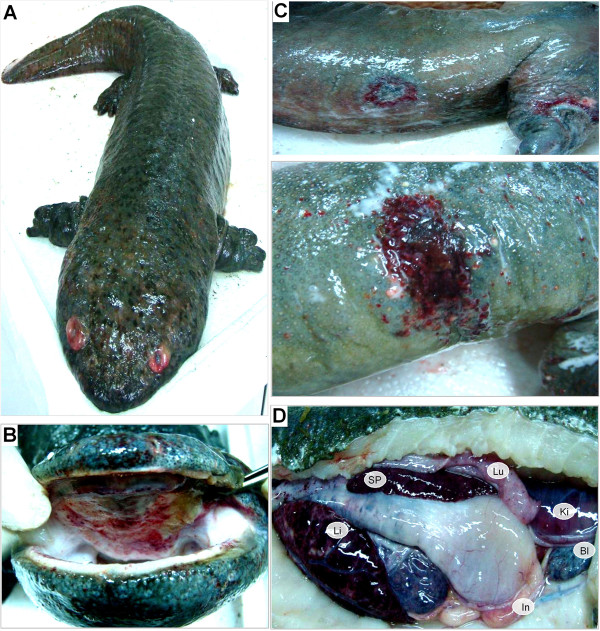
**Main symptoms of the diseased Chinese giant salamanders.** The hyperemic and edematous orbit **(A)**, ecchymotic oral mucosa **(B)**, skin edema and ulcer **(C)**, congestion and swelling in the liver (Li) and spleen (Sp), as well as purplish ecchymoses in the kidney (Ki), lung (Lu), intestines (In) and bladder (Bl) **(D)**, are shown.

Gross examination revealed consistent syndromes among diseased Chinese giant salamanders, including systemic hemorrhage and swelling in any internal or external tissues. As shown in Figure 1, the diseased giant salamanders show hyperemic and edematous orbits (Figure 1A), ecchymotic oral mucosa (Figure 1B), cutaneous erosions and ulcerations (Figure 1C), and extensive hemorrhages on the body surface. Anatomical observations show congestion and swelling in the liver and spleen, petechial lesions and purplish ecchymoses in the kidney, lung, intestines, and bladder (Figure 1D).

**Figure 2:**
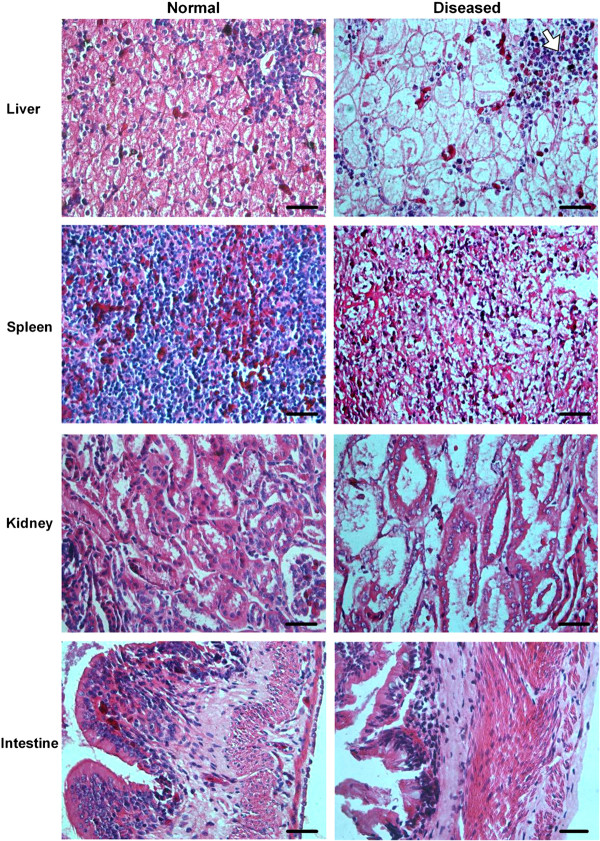
**Histopathological changes in the liver, spleen, kidney and intestine of the diseased Chinese giant salamanders.** The tissue sections from normal (left) and diseased (right) salamanders were stained by hematoxylin and eosin. Various degrees of damage and cell necrosis are present in the diseased liver, spleen, kidney, and intestine. An irregular hemorrhagic patch and sinusoidal congestion is shown by the arrow in the diseased liver tissue. Bar = 200 μm.

Microscopic examination shows various degrees of damage and cell necrosis in tissues from diseased salamanders (Figure 2). Vacuolar degeneration of hepatocytes, focal necrosis areas evidencing pyknosis and karyolysis, and sinusoidal congestion were observed in the liver. Diffuse necrosis of the splenic parenchyma, karyolysis of nuclei, and irregularly shaped cavities were present in the spleen. Severe renal lesions, including degeneration and necrosis of renal tubular epithelia cells, and tubular collapse were frequently observed. The intestinal villi were destroyed and detached, and necrosis of mucous epithelia cells and muscular fibrosis were present in the intestine. No lesions were observed in the corresponding normal tissues (Figure 2).

### Virus isolation and identification

**Figure 3:**
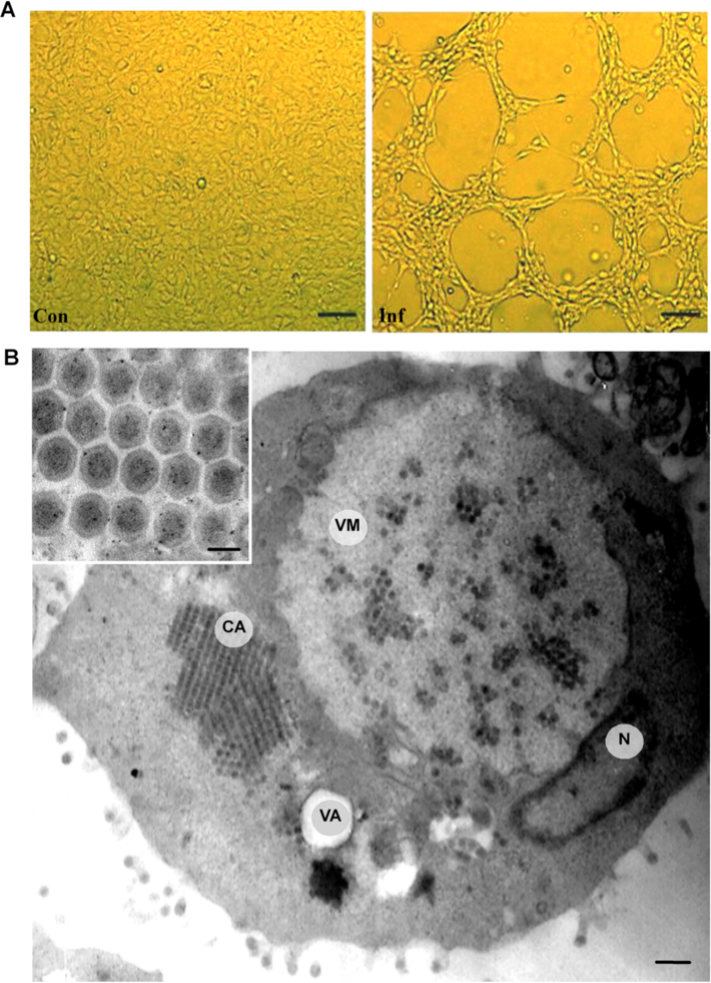
**Light microscopy and electron microscopy observations. (A)** Light micrographs of normal control (Con) and the ADRV infected (Inf) EPC cells. Cytopathic effect (CPE) was observed at 48 h post-infection, Bar = 200 μm. **(B)** Electron micrographs of the ADRV infected cell. A large viromatrix (VM) nearby the nucleus (N) and containing some scattered viral particles, a viral crystalline aggregate (CA) alongside the viromatrix. VA, vacuole Bar = 500 nm. On the upper left corner is an enlarged image, showing the ADRV crystalline aggregate. Bar = 150 nm.

Tissue extracts from diseased salamanders induced cytopathic effect (CPE) in EPC, FHM, GCF, GCO, CHSE and BF-2 cell lines after 24 h to 48 h of incubation. The CPE characteristics in EPC cells included microscopic foci, cell rounding and detachment, and extensive focal plaques, which rapidly progressed to the entire cell sheets with complete destruction occurring within 48 h post infection (Figure 3A). Virus titers in these 6 cell lines ranged from 10^4.5^ to 10^6.5^ TCID_50_mL^-1^ (Table 1). Biophysical and biochemical analysis revealed that the virus was sensitive to heat (56 °C, 30 min), acid (pH 3.0) and alkaline (pH 10.0). Treatment of the virus with either caused significant reduction of its infectivity. The viral infectivity was almost completely inhibited by chloroform and 5-iodo-2-deoxyuridine (IUdR) treatments, indicating that the virus possessed a DNA genome and alipid-containing envelope.

**Table 1:** Different fish cell lines were infected with ADRV.

**Fish cell line**	**Time (h) of first appearance of the cytopathic effect**	**Viral titer (TCID**_**50**_**mL**^**-1**^**)**
*Epithelioma papulosum* cyprini (EPC)	24	10^6.5^
Chinook salmon embryo (CHSE)	24	10^6.5^
Bluegill fry (BF-2)	24	10^6.0^
Grass carp fins (GCF)	36	10^6.0^
Grass carp ovary (GCO)	36	10^5.5^
Fathead minnow (FHM)	48	10^4.5^

Ultrastructural observations of infected EPC cells show features consistent with infections by ranaviruses [30,42,43], including large vitromatrix (VM) for virus factory within the cytoplasm, viral crystalline aggregate (CA), and mature virus particle budding from the cell plasma membrane (Figure 3B). Some cellular changes including nuclear compaction and margination and vacuole formation were also present in the infected cell. The mature virus particles within the crystalline aggregate were relatively uniform in size, with a diameter of about 150 ~ 160 nm. Based on the disease characteristics, species infected, and ultrastructural observations, the newly emerging virus from Chinese giant salamander was therefore identified as *Andrias davidianus* ranavirus (ADRV).

### Pathogenicity of ADRV

To confirm the pathogenicity, ADRV from EPC cell cultures was used to infect healthy Chinese giant salamanders. Some typical hemorrhage and swelling syndromes, identical to the naturally diseased salamanders, were observed from the infected animals after 1 week, and 100% mortality occurred within 3 weeks. The histological changes in the infected tissues were similar to those described above, and virus was re-isolated from the challenged salamanders. These data demonstrate that ADRV is the aetiological agent of the disease, and proved highly pathogenic to Chinese giant salamander.

### General features of ADRV genome

The complete nucleotide sequence of ADRV consists of 106,734 bp, with a G + C content of 55%. Computer-assisted analysis revealed 101 open reading frames (ORFs), which encode putative proteins ranging in size from 44 to 1294 amino acids. All detailed annotation data for the 101 ORFs, such as position, size, predicted function, conserved domain, and their homologous comparisons with other amphibian ranaviruses (RGV, CMTV, FV3, TFV and ATV) are provided in Additional file 2.

**Figure 4:**
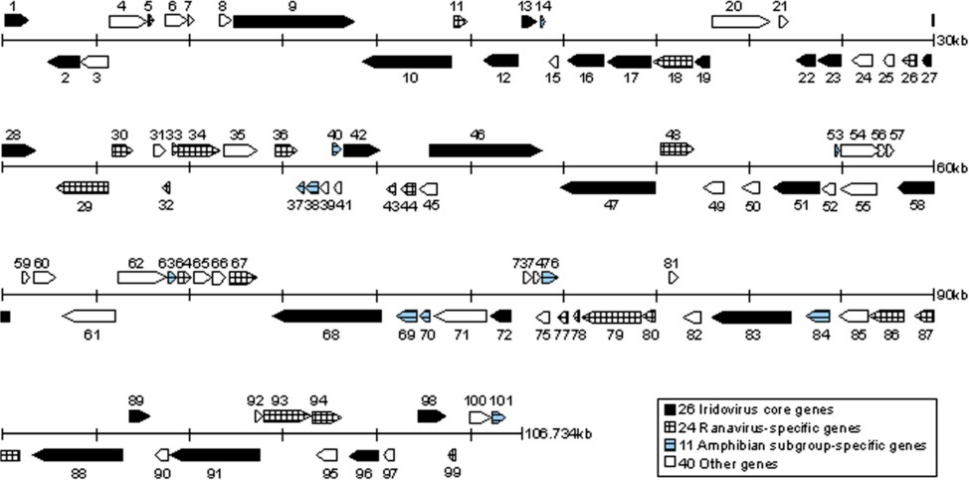
**Schematic diagram of ADRV genome organization.** The ADRV genome is 106 734 bp in size, and contains 101 potential open reading frames (ORFs). The scale is in kilobase pairs. Arrows indicate the size, location, orientation of the ORFs. The ADRV genome contains 26 iridovirus core genes, 24 ranavirus-specific genes, 11 amphibian subgroup-specific genes and 40 other genes.

A schematic diagram of ADRV genome organization is shown in Figure 4. Based on sequence homology to other characterized proteins, 26 ORFs were identified as iridovirus core genes [17], 24 ORFs as ranavirus-specific genes and 11 ORFs as amphibian subgroup-specific genes, whereas 40 other potential genes were unknown for their characterization or function (see Additional file 2).

### Phylogenetic relationship of ADRV and other iridoviruses

**Figure 5:**
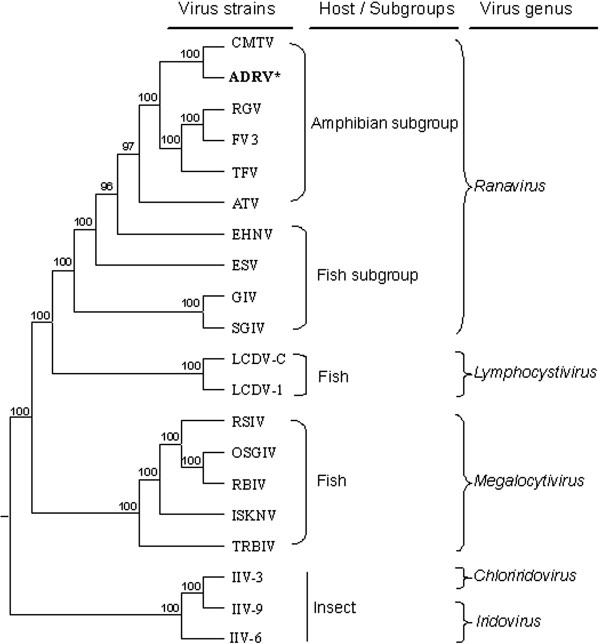
**Phylogenetic tree of 26 iridoviral core protein sequences from 20 completely sequenced iridoviruses.** Consensus bootstrap confidence values are indicated at the nodes of the branches. The host of each virus and viral genus are listed at the right. Two subgroups, such as “Amphibian subgroup” and “Fish subgroup”, were revealed in genus *Ranavirus*. ADRV appears in the amphibian subgroup, and it is most closely related to CMTV, RGV, FV3 and TFV. The sequences used for this analysis are collected in Additional file 1.

A phylogenetic tree was constructed based on the concatenated protein sequences of the 26 iridoviral core genes from 20 completely sequenced iridoviruses (Figure 5). The tree shows that ADRV clustered closely with members of the genus *Ranavirus* in the family *Iridovidae*, and is distantly related to the members from *Lymphocystivirus*, *Megalocytivirus*, *Chloriridovirus* and *Iridovirus*. It was also observed that ranaviruses of the genus *Ranavirus* were separated into two subgroups, the amphibian subgroup including ADRV, CMTV, RGV, FV3, TFV and ATV, and the fish subgroup including EHNV, ESV, GIV and SGIV. ADRV is more closely related to CMTV, RGV FV3 and TFV, which are frog (anuran) ranaviruses, than to ATV, a salamander (urodele) ranavirus.

### Genome sequence comparisons

**Figure 6:**
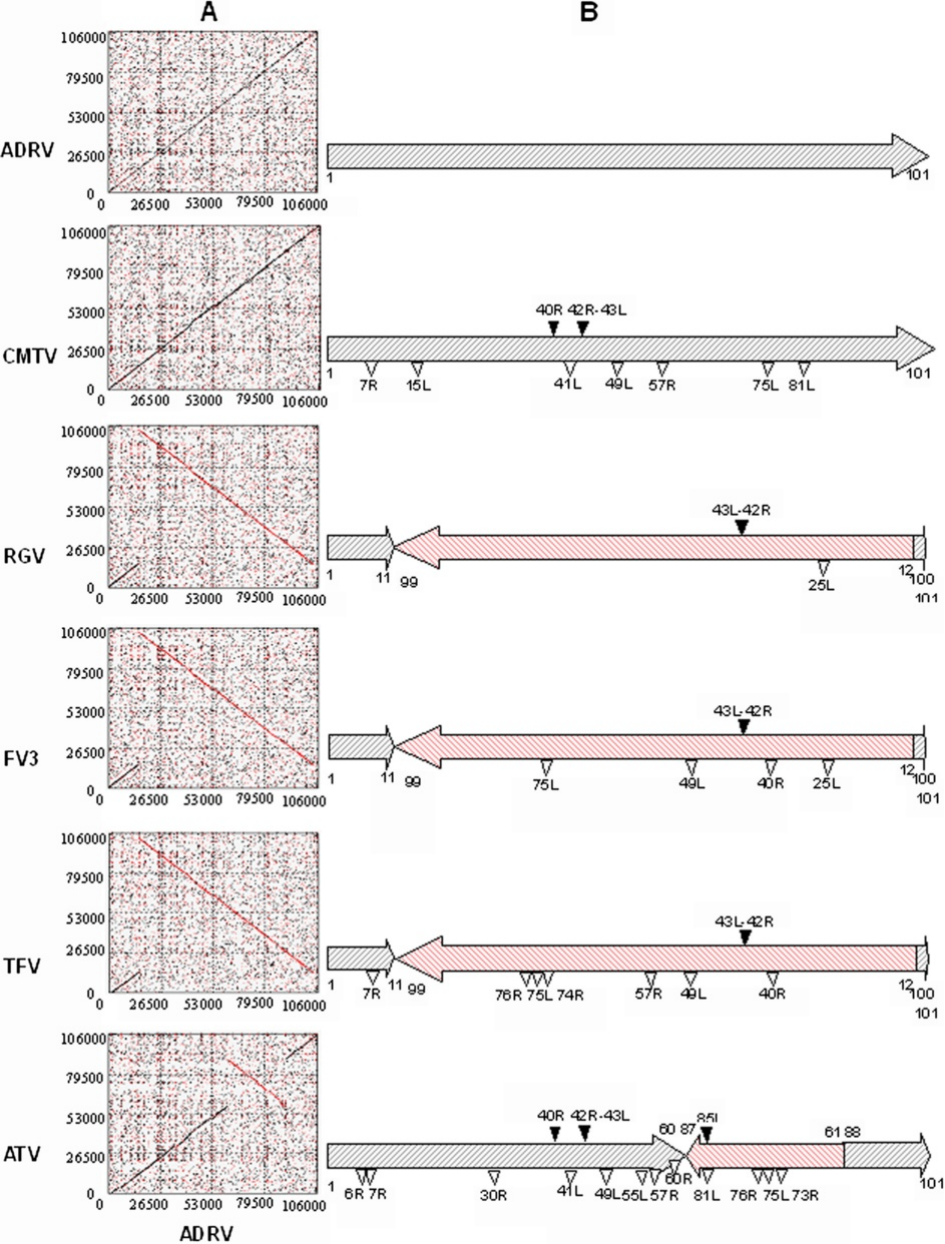
**Dot plot comparisons and genome architecture changes of amphibian subgroup ranaviruses. (A)** Dot plot comparisons between ADRV (horizontal axis) and known amphibian ranaviruses (vertical axis), such as ADRV, CMTV, RGV, FV3, TFV and ATV, respectively. **(B)** Schematic diagram of genome architecture changes relative to ADRV among amphibian subgroup ranaviruses, such as ADRV, CMTV, RGV, FV3, TFV and ATV, respectively. Red hatched arrows indicate genome segment inversion, black triangles show fragment insertion, and blank triangles represent fragment deletion in comparison with the ADRV genome.

To determine the similarity degree of the ADRV genome with those of other amphibian ranaviruses, we carried out dot plot analysis (Figure 6A). Interestingly, a complete colinearity between ADRV and CMTV genomes was revealed as +45° line, and just only a single inversion in the ADRV genome was detected to occur in the segment from 15049 to 104729 in comparison with RGV, FV3 and TFV, and in the segment from 61713 to 90897 compared to ATV.

A comparison of genome architecture and the annotated genes between ADRV and other ranaviruses revealed significant deletion of 6 genes (7R, 15L, 41L, 49L, 57R and 81R in ADRV) in CMTV although they have complete colinearity, whereas only one gene deletion (25L in ADRV) was found in RGV (see Additional file 2). Moreover, two inserted fragments were detected from the CMTV genome, in which a 765 bp fragment was inserted at the corresponding position between 40792 and 40793 of ADRV ORF 40R, and another 879 bp fragment was inserted at the ADRV 42127–42128 position between ORF 42R and 43L. The latter inserted fragment was also observed in the corresponding position of RGV, FV3, TFV and ATV genomes with variable size of 760 bp, 757 bp, 750 bp and 762 bp respectively, whereas the former fragment was only found in the ATV genome, and another fragment of 1643 bp was inserted into ATV genome at the corresponding position between 87890 and 87891 of ADRV ORF 85L (Figure 6B). The statistical analysis shows that ADRV contained 99%, 97%, 94%, 93% and 85% homologous genes in RGV, FV3, CMTV, TFV, and ATV, respectively. Owing to these deletions/insertions, some genes were discovered to exist in certain species of ranaviruses. For examples, 49L exists only in ADRV and RGV, 7R and 57R in ADRV, RGV and FV3, 15L, 41L and 81R in ADRV, RGV, FV3 and TFV, 75L and 76R in ADRV, RGV, CMTV and FV3, and 25L in ADRV, CMTV, TFV and ATV (see Additional file 2).

### Major gene identification for contribution to pathogen virulence and species susceptibility

**Figure 7:**
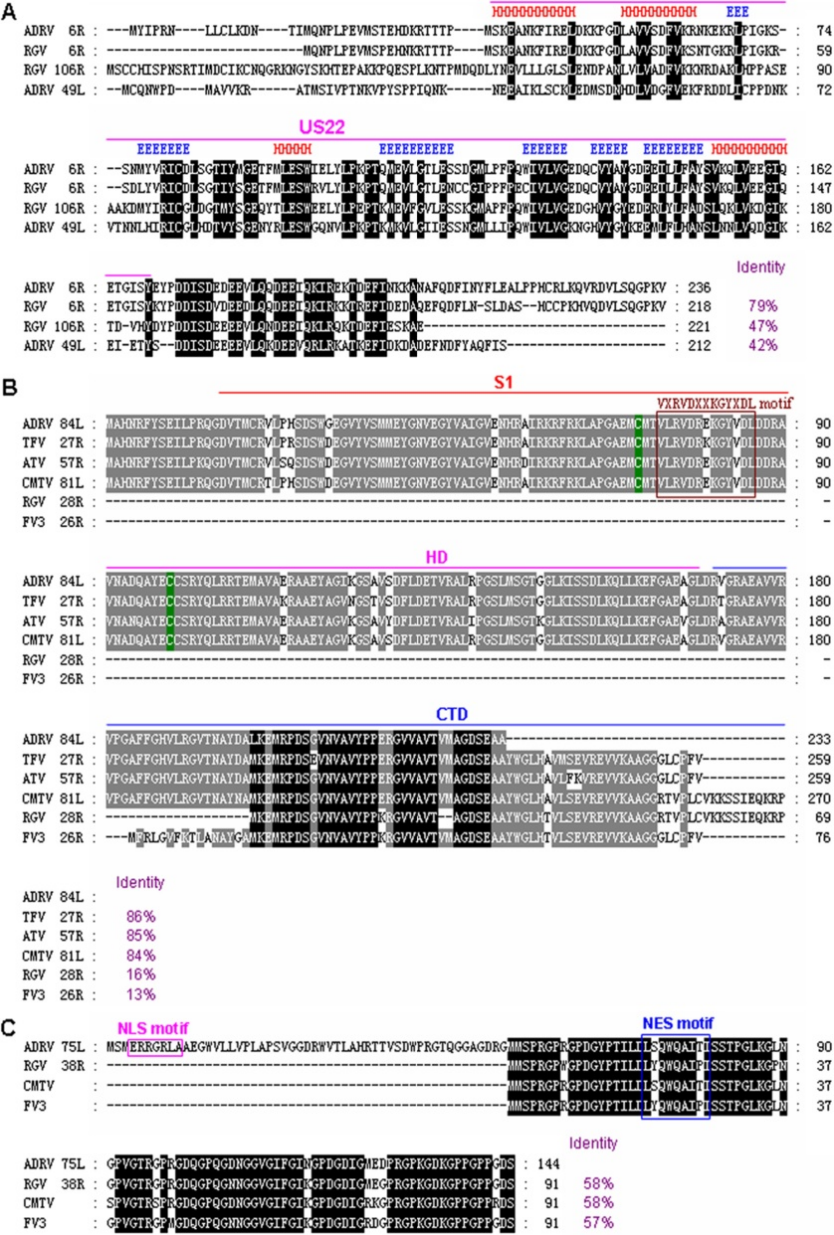
**Amino acid sequence alignments of three major genes among ADRV and other ranaviruses. (A)** Multiple alignments of the deduced two duplicate US22 family-like proteins in ADRV (6R, 49L) and RGV (6R, 106R). The secondary structure of US22 domain that is comprised of four conserved α-helices (red H) and six β-strands (blue E) was shown above the alignments, and the completely conserved residues are indicated by black shaded regions. **(B)** Multiple alignments of ADRV 84L and its homologues in other known ranaviruses, such as TFV, ATV, CMTV, RGV and FV3. The N-terminal PKR-binding domain (S1), central helical domain (HD) and C-terminal domain (CTD) are shown above the alignments, and two cysteines are indicated by green. The VXRVDXXKGYXDL motif is marked by a box. The black shaded regions indicate completely conserved residues, while the grey shaded regions are partially conserved residues with greater than 80% identity. **(C)** Multiple alignments of ADRV 75L and its homologues in RGV, CMTV and FV3. The homologous regions for ADRV 75L in CMTV (nucleotides 77077–77352) and FV3 (nucleotides 41633–41908) were translated into amino acid sequences and used in the alignments. The conserved residues are indicated by black shaded regions. The nuclear localization signal (NLS) motif is marked by the purple box, and the nuclear export signal (NES) motif by the blue box.

Gene annotation and comparative analysis of ADRV with other ranaviruses revealed several highly variable and significantly functional genes in these ranavirus, which might be related to pathogen virulence and species susceptibility. For example, two duplicate genes encoding US22 family-like proteins were found in ADRV (6R and 49L) and RGV (6R and 106R), whereas only one homologue was observed in CMTV, FV3 and TFV (see Additional file 2). The deduced amino acid sequence of ADRV 6R shows 42%, 47%, and 79% identity to ADRV 49L, RGV 106R and RGV 6R respectively (Figure 7A), suggesting that these two duplicate genes in ADRV have been highly diversified from each other.

Another viral pathogenesis and virulence-related gene vIF2α, the homologue of eukaryotic translation initiation factor 2 alpha (eIF2α), was also found to be highly variable among ADRV and other ranaviruses. The N-terminal PKR-binding domain (S1) and central helical domain (HD) of vIF2α were highly conserved among ADRV 84L, TFV 27R, ATV 57R and CMTV 81L, but completely absent in RGV 28R and FV3 26R, and only 69 and 76 amino acids of the C-terminal domain (CTD) remained in RGV and FV3 vIF2α (Figure 7B). In addition, the C-terminal region was variable among these vIF2α, leading to their size and identity differences, in which RGV 28R and FV3 26R have only 16% and 13% identities to ADRV 84L.

Moreover, we were fascinated by another novel gene ADRV 75L, which also existed in RGV (38R), CMTV (nucleotides 77077–77352) and FV3 (nucleotides 41633–41908). However, the corresponding homologous regions for ADRV 75L were not previously annotated in CMTV and FV3. ADRV 75L encodes a peptide of 144 amino acids, and contains a nuclear localization signal (NLS) motif and a nuclear export signal (NES) motif, whereas its homologues in RGV, CMTV and FV3 lack a 53 amino acid N-terminal sequence and the NLS motif, and have only a NES motif (Figure 7C). The deduced amino acid sequence of ADRV 75L shows 57-58% identities to the homologues in RGV, CMTV and FV3.

## Discussion

Ranaviruses are known to be agents of emerging infectious disease that have raised global concern. Chinese giant salamanders have been hit by viral epidemic diseases in natural habitats or on farms since 2010 [22,23]. The universal symptoms include systemic hemorrhage and serious swelling. In this study, we isolated and identified a novel ranavirus (ADRV) from diseased Chinese giant salamanders, which could cause typical CPE in different fish cell lines with yielding high virus titers and displayed severe pathogenicity in vivo. We also determined and characterized the complete genome sequence of ADRV. This is the second completely sequenced ranavirus isolate from urodele; the first one was ATV [44]. Based on genome size, gene content and phylogenetic analysis, ADRV should belong to the Amphibian subgroup of ranaviruses, and is more closely related to CMTV, RGV, FV3 and TFV (frog ranaviruses) than to ATV (salamander ranavirus).

Dot plot analysis shows that ADRV shared complete colinearity with a toad ranavirus CMTV, and a single genomic inversion was detected in ADRV compared to RGV, FV3, TFV and ATV (Figure 6). Significantly, the inversion does not involve any loss of genetic information, and the inversion position is an absolute coincidence found previously in CMTV [20]. However, the detailed genome comparisons of ADRV and other ranaviruses revealed significant deletion of 6 genes (7R, 15L, 41L, 49L, 57R and 81R in ADRV) in CMTV, whereas only one gene deletion (25L in ADRV) was observed in RGV. Moreover, two inserted fragments were detected in the CMTV genome, while only one inserted fragment was observed in the corresponding position of RGV, FV3, TFV and ATV genomes. Homologous gene comparison analysis shows that ADRV contained 99%, 97%, 94%, 93% and 85% homologues in RGV, FV3, CMTV, TFV and ATV respectively, and the largest homologues existed between ADRV and RGV (see Additional file 2). In addition, two duplicate genes encoding US22 family-like proteins were found in ADRV (6R and 49L) and RGV (6R and 106R), whereas only one homologue was observed in CMTV, FV3 and TFV. Furthermore, RGV is a pathogenic agent that causes lethal disease prevailing in China [2,13,14,19], while CMTV is a disease agent geographically confined to the European continent [20]. Based on this information and the fact that the Chinese giant salamander is endemic to mainland China, we speculate that ADRV and RGV might occupy a common or closer evolutionary emergence in these ranaviruses.

We have been wondering about where ADRV emerges from and why ADRV causes a lethal disease and results in epidemic outbreaks in Chinese giant salamanders? The current data provide significant genetic evidence for evolutionary emergence of ranaviruses. Significant genomic changes including segment inversion, and fragment insertion and deletion were observed in comparing ADRV to other amphibian ranaviruses. Previous studies have shown that the inversions between different ranavirus isolates reflect the high recombination rate of ranaviruses, and these genomic rearrangements may create novel, more pathogenic viral strains as some genes are disrupted or added [18,44]. Moreover, several major virulence-related gene variations were also identified. For examples, two duplicate genes encoding US22 family-like proteins are highly diversified in ADRV (6R and 49L) and RGV (6R and 106R), and the identity percentages between amino acid sequences ranged from 42% to 79%. US22 family genes have been reported in various herpesviruses, adenoviruses, poxviruses and iridoviruses [45], and most of the family members are associated with viral replication and pathogenesis [46-48], suggesting that the highly diversified US22 family-like genes might contribute to viral pathogenesis and species susceptibility of ADRV and RGV. Another highly variable virulence-related gene is vIF2α, in which two functional domains (S1 and HD) are completely absent in RGV and FV3. There are only 69 and 76 amino acids remaining in part of the C-terminal domain. Recently, the role of vIF2α in blocking the antiviral effects of cellular PKR has been confirmed in some ranaviruses [49,50], and the truncated vIF2α in FV3 was also demonstrated to be involved in viral pathogenesis [51]. Thus, the variable vIF2αs in ranaviruses might be associated with viral pathogenesis and host susceptibility. The third major gene variation is a novel gene. In ADRV, the novel 75L gene contains both motifs of NLS and NES, whereas its homologues in RGV, FV3 and CMTV lack a 53 amino acid N-terminal sequence and the NLS motif, and has only a NES motif. NLS motifs have been suggested to be important for quick import and export of viral nucleoprotein [52,53] and for efficient viral protein synthesis [54,55], and the nuclear localization role has been demonstrated in RGV 50L gene with an NLS motif [56]. Perhaps, the novel 75L gene with NLS and NES motifs might be involved in viral protein synthesis and transport during ADRV infection, and might be a strongly virulent gene. Indeed, genome architecture changes and major gene variations are the raw material basis for evolutionary emergence, and major gene variations have been shown to determine the pathogenicity of viruses [57,58]. Therefore, our current findings suggest that ADRV might emerge from a common ancestor of amphibian-subgroup ranaviruses, in which the corresponding genetic change routes through genomic changes include segment inversion, fragment insertion and deletion, and some major virulence-related gene variations.

In conclusion, we show that ADRV is the etiologic agent for lethal epidemic diseases in Chinese giant salamanders. Genome characterization and comparison analysis indicates that ADRV should be a new member of the Amphibian subgroup of ranaviruses, and that genomic architecture changes and several gene variations may contribute to evolutionary emergence of ADRV. Since ranavirus can infect a wide range of hosts, and its spread has been increased with global air travel and anthropogenic movement of animals [18], further research into exploring the ecological and anthropogenic mechanisms of emergence of ADRV-caused diseases will be important for controlling this emerging pathogen.

## Competing interests

The authors declare that they have no competing interests.

## Authors’ contributions

QYZ and JFG conceived and designed the experiments; ZYC, XCG, CP and YJH performed the experiments; QYZ, JFG and ZYC analyzed the data; ZYC, YJH, JFG and QYZ contributed reagents/materials/analysis tools; JFG, QYZ and ZYC wrote the paper. All authors read and approved the final manuscript.

## Supplementary Material

Additional file 1 **Information of 21 completely sequenced iridoviruses.** Summary of genomic sequence information of 21 iridovirus isolates from five genera within the family *Iridoviridae*. Click here for file

Additional file 2 **Characterization of predicted open reading frames (ORFs) of ADRV.** The detailed annotation data for ADRV ORFs, such as position, size, predicted function, conserved domain, and their homologous comparisons with other amphibian ranaviruses are shown. Click here for file
