# Supplementary material for: Genome architecture changes and major gene variations of Andrias davidianus ranavirus (ADRV)
Source: Vet Res. 2013 Oct 21;44(1):101. doi: 10.1186/1297-9716-44-101 (PMC4015033; doi:10.1186/1297-9716-44-101)
Supplement: Additional file 1 — Information of 21 completely sequenced iridoviruses. Summary of genomic sequence information of 21 iridovirus isolates from five genera within the family Iridoviridae. [file 1297-9716-44-101-S1.doc]

**Additional file 1 Information of 21 completely sequenced iridoviruses.**

| **Genus and virus** | **Full name** | **Known host** | **Isolation**  **region and time** | **Genome size (kb)** | **GC (%)** | **Potential**  **ORFs** | **ORF size**  **(aa)** | **GenBank**  **accession no.** |
| --- | --- | --- | --- | --- | --- | --- | --- | --- |
| *Ranavirus* |  |  |  |  |  |  |  |  |
| ADRV | *Andrias davidianus* ranavirus | Chinese giant salamander | China, 2012 | 106.734 | 55 | 101 | 49–1294 | KC865735 |
| FV3 | Frog virus 3 | Frog | America, 1966 | 105.903 | 55 | 98 | 50–1293 | AY548484 |
| EHNV | Epizootic hematopoietic necrosis virus | Fish | Australia, 1986 | 127.011 | 54 | 100 | 49–1303 | FJ433873 |
| ESV | European sheatfish ranavirus | Fish | Europe, 1989 | 127.732 | 54 | 136 | 40–1298 | JQ724856 |
| RGV | *Rana grylio* virus | Frog | China, 1995 | 105.791 | 55 | 106 | 41–1294 | JQ654586 |
| SGIV | Singapore grouper iridovirus | Fish | Singapore,1998 | 140.131 | 48 | 162 | 41–1268 | AY521625 |
| GIV | Grouper iridovirus | Fish | Taiwan, 2000 | 139.793 | 49 | 120 | 62–1268 | AY666015 |
| TFV | tiger frog virus | Frog | China, 2002 | 105.057 | 55 | 105 | 40–1294 | AF389451 |
| ATV | *Ambystoma tigrinum* virus | Salamander | America, 2003 | 106.332 | 54 | 96 | 32–1294 | AY150217 |
| CMTV | Common midwife toad ranavirus | Toad | Europe, 2007 | 106.878 | 55 | 104 | 46–1321 | JQ231222 |
| STIV | Soft-shelled turtle iridovirus | Turtle | China, 1999 | 105.890 | 55 | 105 | 40-1294 | EU627010 |
| *Lymphocystivirus* |  |  |  |  |  |  |  |  |
| LCDV-1 | Lymphocystis disease virus 1 | Fish | Red Sea, 1962 | 102.653 | 29 | 110 | 40–1199 | L63545 |
| LCDV-C | Lymphocystis disease virus-China | Fish | China, 2000 | 186.250 | 27 | 240 | 40–1193 | AY380826 |
| *Megalocytivirus* |  |  |  |  |  |  |  |  |
| RSIV | Red seabream iridovirus | Fish | Japan, 1992 | 112.414 | 53 | 93 | 86–1309 | AB104413 |
| ISKNV | Infectious spleen and kidney necrosis virus | Fish | China, 1998 | 111.362 | 55 | 124 | 40–1208 | AF371960 |
| RBIV | Rock bream iridovirus | Fish | South Korea,  2000 | 112.080 | 53 | 118 | 50–1253 | AY532606 |
| TRBIV | Turbot reddish body iridovirus | Turbot | China, 2004 | 110.104 | 55 | 115 | 40–1168 | GQ273492 |
| OSGIV | Orange-spotted grouper iridovirus | Fish | China, 2005 | 112.636 | 54 | 121 | 40–1168 | AY894343 |
| *Iridovirus* |  |  |  |  |  |  |  |  |
| IIV-6 | Invertebrate iridescent virus type 6 | Insect | Japan, 1966 | 212.482 | 29 | 243 | 40–2432 | AF303741 |
| IIV-9 | Invertebrate iridescent virus type 9 | Insect | New Zealand,  1972 | 205.791 | 31 | 191 | 50–2051 | GQ918152 |
| *Chloriridovirus* |  |  |  |  |  |  |  |  |
| IIV-3 | Invertebrate iridescent virus type 3 | Insect | America, 1965 | 191.132 | 48 | 126 | 60–1377 | DQ643392 |
