# Supplementary material for: Genome architecture changes and major gene variations of Andrias davidianus ranavirus (ADRV)
Source: Vet Res. 2013 Oct 21;44(1):101. doi: 10.1186/1297-9716-44-101 (PMC4015033; doi:10.1186/1297-9716-44-101)
Supplement: Additional file 2 — Characterization of predicted open reading frames (ORFs) of ADRV. The detailed annotation data for ADRV ORFs, such as position, size, predicted function, conserved domain, and their homologous comparisons with other amphibian ranaviruses are shown. [file 1297-9716-44-101-S2.doc]

**Additional file 2 Characterization of predicted open reading frames (ORFs) of ADRVa.**

| **ORF** | **Nucleotide**  **position** | **AA** | **kDa** | **IP** | **Predicted function/conserved domain** | **RGV^c^** | | | **CMTV^c^** | | | **FV3^c^** | | | **TFV^c^** | | | **ATV^c^** | | |
| --- | --- | --- | --- | --- | --- | --- | --- | --- | --- | --- | --- | --- | --- | --- | --- | --- | --- | --- | --- | --- |
|  |  |  |  |  |  | **ORF** | **AA** | **% ID^d^** | **ORF** | **AA** | **%ID^d^** | **ORF** | **AA** | **%ID^d^** | **ORF** | **AA** | **%ID^d^** | **ORF** | **AA** | **%ID^d^** |
| 1R^b^ | 16-786 | 256 | 29.73 | 9.36 | Replicating factor | 1R | 256 | 98.0 | 1R | 256 | 98.8 | 1R | 256 | 98.8 | 105R | 256 | 98.4 | 91R | 256 | 96.1 |
| 2L^b^ | 1502-2479 | 325 | 35.19 | 8.10 | Myristylated membrane protein, DUF230 poxvirus of unknown function, TM | 2L | 323 | 95.4 | 2L | 337 | 92.6 | 2L | 320 | 96.0 | 2L | 237 | 67.7 | 1L | 303 | 85.2 |
| 3L | 2517-3392 | 291 | 32.60 | 7.74 |  | 3L | 292 | 97.9 | 3L | 279 | 92.8 | NA |  |  | NA |  |  | 2L | 279 | 92.4 |
| 4R | 3386-4600 | 404 | 44.27 | 6.02 |  | 4R | 404 | 95.0 | 4R | 404 | 96.3 | 3R | 438 | 87.4 | 4R | 404 | 96.3 | 3R | 404 | 94.3 |
| 5R | 4640-4822 | 60 | 6.64 | 9.54 | TM | 5R | 60 | 96.7 | 5R | 60 | 96.7 | 4R | 60 | 96.7 | 5R | 60 | 91.7 | 4R | 60 | 90.0 |
| 6R | 5211-5921 | 236 | 27.30 | 4.55 | FPV ORF250, herps virus US22 family like protein | 6R | 218 | 77.1 | 6R | 200 | 77.5 | 5R | 204 | 72.9 | 6R | 200 | 69.5 | – |  |  |
| 7R | 5893-6111 | 72 | 8.39 | 5.56 |  | 7R | 83 | 84.3 | – |  |  | 6R | 75 | 94.7 | – |  |  | – |  |  |
| 8R | 6902-7288 | 128 | 13.71 | 10.15 |  | 8R | 128 | 94.5 | NA |  |  | 7R | 128 | 95.3 | NA |  |  | – |  |  |
| 9R^b^ | 7380-11264 | 1294 | 140.95 | 6.51 | DNA-dependent RNA polymerase II largest subunit | 9R | 1294 | 98.5 | 8R | 1321 | 96.9 | 8R | 1293 | 98.5 | 8R | 1294 | 98.7 | 6R | 1294 | 98.1 |
| 10L^b^ | 11627-14473 | 948 | 106.30 | 8.19 | NTPase, SNF2 family N-terminal domain, DEAD-like helicases superfamily | 10L | 948 | 98.4 | 9L | 948 | 98.8 | 9L | 948 | 98.7 | 9L | 948 | 97.8 | 7L | 948 | 98.3 |
| 11R | 14489-14902 | 137 | 14.94 | 9.69 | TM | 11R | 137 | 95.6 | 10R | 137 | 99.3 | 10R | 137 | 95.6 | 10R | 137 | 97.1 | 8R | 137 | 97.8 |
| 12L^b^ | 15542-16633 | 363 | 40.57 | 7.33 | DNA repair protein RAD2 | 102R | 363 | 99.2 | 12L | 364 | 98.4 | 95R | 363 | 98.9 | 101R | 390 | 91.8 | 10L | 364 | 97.3 |
| 13R^b^ | 16726-17193 | 155 | 17.83 | 9.45 | P8.141C-like protein, TM | 101L | 155 | 98.1 | 13R | 155 | 98.7 | 94L | 155 | 98.1 | 100L | 155 | 98.1 | 11R | 155 | 96.1 |
| 14R | 17304-17471 | 55 | 5.68 | 3.86 |  | 100L | 71 | 73.2 | 14R | 71 | 74.6 | 93L | 55 | 96.4 | 99L | 55 | 94.5 | 12R | 51 | 81.8 |
| 15L | 17661-17924 | 87 | 10.12 | 10.05 |  | 99R | 101 | 53.5 | – |  |  | 92R | 79 | 82.8 | 98R | 101 | 73.5 | – |  |  |
| 16L^b^ | 18227-19414 | 395 | 45.42 | 6.05 | Immediate early protein ICP-46 | 98R | 395 | 97.5 | 15L | 395 | 97.2 | 91R | 395 | 97.5 | 97R | 395 | 95.7 | 13L | 395 | 94.9 |
| 17L^b^ | 19538-20929 | 463 | 49.97 | 5.95 | Major capsid protein | 97R | 463 | 98.3 | 16L | 463 | 99.8 | 90R | 463 | 97.8 | 96R | 463 | 97.8 | 14L | 463 | 97.8 |
| 18L | 21022-22266 | 414 | 47.06 | 9.77 |  | 96R | 381 | 85.8 | 17L | 359 | 84.1 | 89R | 388 | 88.6 | 95R | 398 | 89.4 | 15L | 424 | 86.1 |
| 19L^b^ | 22334-22786 | 150 | 16.58 | 8.95 | Thiol oxidoreductase, Erv1/Alr family | 95R | 150 | 98.7 | 18L | 150 | 98.7 | 88R | 150 | 98.0 | 94R | 150 | 97.3 | 16L | 150 | 95.3 |
| 20R | 22819-24636 | 605 | 65.28 | 10.13 |  | 94L | 593 | 95.9 | 19R | 599 | 97.2 | 87L | 605 | 98.2 | 93L | 605 | 95.2 | 17R | 608 | 95.2 |
| 21R | 24989-25270 | 93 | 10.54 | 9.87 |  | 93L | 81 | 37.6 | 20R | 71 | 52.7 | 86L | 61 | 49.5 | 92L | 97 | 48.5 | 18R | 125 | 60.0 |
| 22L^b^ | 25617-26204 | 195 | 22.18 | 5.06 | Deoxynucleoside kinase/thymidine kinase | 92R | 195 | 97.9 | 21L | 195 | 98.5 | 85R | 195 | 97.4 | 91R | 195 | 99.5 | 19L | 195 | 95.4 |
| 23L^b^ | 26279-27016 | 245 | 26.14 | 7.67 | Proliferating cell nuclear antigen | 91R | 245 | 98.0 | 22L | 245 | 99.2 | 84R | 245 | 98.4 | 90R | 245 | 97.6 | 20L | 260 | 91.2 |
| 24L | 27418-28062 | 214 | 24.85 | 8.95 | Cytosine DNA methyltransferase | 90R | 214 | 97.2 | 23L | 214 | 99.1 | 83R | 214 | 97.7 | 89R | 214 | 97.7 | 21L | 214 | 96.3 |
| 25L | 28429-28716 | 95 | 10.81 | 5.19 | Thymidylate synthase | – |  |  | 24L | 95 | 96.8 | – |  |  | 88R | 124 | 72.6 | 22L | 95 | 95.8 |
| 26L | 29014-29487 | 157 | 17.43 | 6.73 | Immediate early protein ICP-18 | 89R | 157 | 89.2 | 25L | 157 | 95.5 | 82R | 157 | 89.2 | 87R | 157 | 92.4 | 23L | 157 | 94.3 |
| 27L^b^ | 29616-29894 | 92 | 10.45 | 8.09 | Transcription elongation factor SII | 88R | 92 | 96.7 | 26L | 92 | 98.9 | 81R | 92 | 97.8 | 86R | 92 | 97.8 | 24L | 92 | 98.9 |
| 28R^b^ | 29955-31073 | 372 | 40.61 | 8.61 | Ribonuclease III | 87L | 371 | 99.2 | 27R | 372 | 99.2 | 80L | 371 | 99.2 | 85L | 371 | 98.4 | 25R | 373 | 97.1 |
| 29L | 31697-33373 | 558 | 61.71 | 8.51 | ATPase-dependent protease | 86R | 572 | 93.0 | 28L | 572 | 94.9 | 79R | 572 | 93.2 | 84R | 572 | 93.2 | 26L | 576 | 86.8 |
| 30R | 33510-34148 | 212 | 24.16 | 5.46 |  | 85L | 224 | 91.1 | 29R | 229 | 89.1 | 78L | 212 | 95.8 | 83L | 224 | 88.8 | – |  |  |
| 31R | 34848-35195 | 115 | 12.84 | 8.91 |  | 84L | 115 | 100.0 | 30R | 115 | 98.3 | 77L | 115 | 100.0 | 82L | 115 | 99.1 | 27R | 115 | 96.5 |
| 32L | 35192-35413 | 73 | 8.00 | 10.05 |  | 83R | 73 | 95.9 | 31L | 73 | 97.3 | 76R | 73 | 97.3 | 81R | 73 | 98.6 | 28L | 73 | 94.5 |
| 33R | 35476-35730 | 84 | 9.26 | 7.04 | LITAF/PIG7 possible membrane associated motif in LPS-induced tumor necrosis factor alpha factor (LITAF), TM | 82L | 84 | 97.6 | 32R | 84 | 98.8 | 75L | 84 | 98.8 | 80L | 94 | 95.2 | 29R | 84 | 92.9 |
| 34R | 35625-36968 | 447 | 47.86 | 11.13 |  | 81L | 364 | 75.2 | 33R | 393 | 86.6 | 74L | 370 | 79.2 | 79L | 330 | 72.0 | 30R | 393 | 82.3 |
| 35R | 37109-38161 | 350 | 39.24 | 8.08 | NTPase/helicase | 80L | 324 | 90.6 | 34R | 350 | 95.1 | 73L | 324 | 89.4 | 78L | 324 | 88.9 | 31R | 330 | 90.9 |
| 36R | 38717-39433 | 238 | 26.21 | 8.95 |  | 79L | 238 | 97.1 | 35R | 238 | 97.5 | 72L | 238 | 95.8 | 77L | 238 | 96.6 | 32R | 107 | 41.6 |
| 37L | 39490-39723 | 77 | 8.34 | 4.63 |  | 78R | 77 | 94.9 | 36L | 77 | 97.4 | 71R | 77 | 96.2 | 76R | 77 | 94.9 | 34L | 78 | 87.2 |
| 38L | 39763-40137 | 124 | 13.39 | 10.74 |  | 77R | 124 | 96.8 | 37L | 124 | 100.0 | 70R | 124 | 98.4 | 75R | 131 | 92.4 | NA |  |  |
| 39L | 40155-40421 | 88 | 9.37 | 7.88 | TM | 76R | 88 | 97.7 | 38L | 88 | 97.7 | 69R | 88 | 97.7 | 74R | 88 | 96.6 | 35L | 88 | 94.3 |
| 40R | 40490-40813 | 107 | 11.46 | 8.71 |  | 75L | 88 | 66.4 | 39R | 154 | 55.8 | – |  |  | – |  |  | 37R | 252 | 32.9 |
| 41L | 40553-40822 | 89 | 9.24 | 8.02 |  | 74R | 95 | 70.5 | – |  |  | 68R | 95 | 69.5 | 72R | 65 | 29.2 | – |  |  |
| 42R^b^ | 40858-42021 | 387 | 43.87 | 5.00 | Ribonucleotide reductase small subunit | 73L | 387 | 98.4 | 40R | 387 | 100.0 | 67L | 387 | 98.4 | 71L | 387 | 97.7 | 38R | 387 | 99.0 |
| 43L | 42424-42711 | 95 | 10.41 | 4.91 | Interleukin-1 beta convertase precursor, caspase activation and recruitment domain (CARD), COP protein | 68R | 95 | 94.7 | 43L | 95 | 92.6 | 64R | 95 | 92.6 | 69R | 87 | 78.9 | 40L | 95 | 91.6 |
| 44L | 42806-43300 | 164 | 17.37 | 5.23 | dUTPase | 67R | 164 | 98.8 | 44L | 164 | 98.8 | 63R | 164 | 97.6 | 68R | 164 | 97.0 | 42L | 145 | 86.6 |
| 45L | 43419-43955 | 178 | 19.57 | 3.99 |  | 66R | 178 | 98.9 | 45L | 180 | 97.2 | NA |  |  | NA |  |  | NA |  |  |
| 46R^b^ | 43681-47346 | 1221 | 133.21 | 7.50 | DNA-dependent RNA polymerase II second largest subunit domain 6, 7, 3, beta subunit | 65L | 1221 | 98.8 | 46R | 1217 | 98.4 | 62L | 1221 | 99.1 | 65L | 1219 | 97.7 | 43R | 1221 | 97.2 |
| 47L^b^ | 47971-51012 | 1013 | 114.61 | 7.96 | DNA polymerase | 63R | 1013 | 98.5 | 47L | 1013 | 98.8 | 60R | 1013 | 98.6 | 63R | 1013 | 98.6 | 44L | 1013 | 97.5 |
| 48R | 51171-52229 | 352 | 40.12 | 8.47 |  | 62L | 352 | 94.3 | 48R | 352 | 94.3 | 59L | 352 | 94.3 | 62L | 352 | 92.6 | 45R | 352 | 93.5 |
| 49L | 52630-53268 | 212 | 24.37 | 4.65 | Herpes virus US22 family like protein | 106R | 221 | 47.6 | – |  |  | – |  |  | – |  |  | – |  |  |
| 50L | 53849-54403 | 184 | 20.49 | 8.75 |  | 61R | 184 | 98.9 | 49L | 184 | 97.8 | NA |  |  | 61R | 184 | 97.3 | 46L | 184 | 96.7 |
| 51L^b^ | 54854-56350 | 498 | 53.52 | 6.74 | Phosphotransferase, serine/threonine protein kinases | 60R | 498 | 98.6 | 51L | 498 | 97.8 | 57R | 498 | 98.6 | 59R | 498 | 97.8 | 47L | 498 | 96.4 |
| 52L | 56391-56795 | 134 | 15.28 | 6.53 |  | 59R | 134 | 97.8 | 52L | 134 | 97.8 | NA |  |  | 58R | 134 | 97.8 | 48L | 134 | 96.3 |
| 53R | 56832-56981 | 49 | 5.21 | 4.49 | TM | 58L | 49 | 95.9 | 53R | 49 | 100.0 | NA |  |  | 57L | 49 | 98.0 | 49R | 49 | 98.0 |
| 54R | 56989-58284 | 431 | 47.24 | 9.44 | Helicase-like protein, DEXDc superfamily | 56L | 431 | 97.7 | 54R | 431 | 98.1 | 55L | 431 | 97.2 | 56L | 431 | 98.4 | 50R | 431 | 96.3 |
| 55L | 57001-58140 | 379 | 40.31 | 10.66 |  | 57R | 379 | 95.8 | 54L | 379 | 98.2 | 55R | 379 | 96.0 | NA |  |  | – |  |  |
| 56R | 58197-58517 | 106 | 11.51 | 10.02 |  | 55L | 106 | 97.2 | 55R | 130 | 78.5 | NA |  |  | NA |  |  | NA |  |  |
| 57R | 58322-58552 | 76 | 8.79 | 9.77 | Nuclear calmodulin-binding protein | 54L | 76 | 98.7 | – |  |  | 54L | 76 | 100.0 | – |  |  | – |  |  |
| 58L^b^ | 58719-60287 | 522 | 54.77 | 6.15 | Lipid membrane protein of large eukaryotic DNA viruses, LIR_F9L, TM | 53R | 522 | 99.2 | 56L | 522 | 99.2 | 53R | 522 | 99.6 | 55R | 522 | 99.0 | 51L | 526 | 96.0 |
| 59R | 60623-60874 | 83 | 9.23 | 9.94 | 3-beta-hydroxysteroid dehydrogenase | 52L | 355 | 19.4 | 57R | 355 | 19.7 | 52L | 355 | 19.4 | 54L | 355 | 18.9 | 52R | 53 | 50.6 |
| 60R | 60969-61682 | 237 | 26.62 | 8.83 | 3-beta-hydroxysteroid dehydrogenase | 52L | 355 | 65.9 | 57R | 355 | 65.9 | 52L | 355 | 65.6 | 54L | 355 | 65.6 | – |  |  |
| 61L | 61939-63624 | 561 | 61.57 | 5.81 |  | 51R | 561 | 97.9 | 58L | 561 | 98.4 | 51R | 561 | 97.9 | 53R | 561 | 97.9 | 76R | 561 | 96.4 |
| 62R | 63704-65230 | 508 | 56.32 | 5.23 | SAP domain | 50L | 499 | 89.8 | 59R | 503 | 96.1 | 49L | 249 | 47.6 | 51L | 194 | 37.0 | 75L | 513 | 86.7 |
| 63R | 65278-65589 | 103 | 11.69 | 9.13 |  | 49L | 83 | 75.7 | 60R | 103 | 95.1 | 48L | 83 | 75.7 | 50L | 83 | 76.7 | p78 | 127 | 70.9 |
| 64R | 65592-66008 | 138 | 15.57 | 6.12 |  | 48L | 138 | 98.6 | 61R | 138 | 97.8 | 47L | 138 | 98.6 | 49L | 138 | 97.8 | 73L | 138 | 93.5 |
| 65R | 66133-66642 | 169 | 18.45 | 12.5 | Neurofilament triplet H1-like protein | 47L | 144 | 74.0 | 62R | 155 | 76.3 | 46L | 81 | 39.1 | 48L | 152 | 73.4 | 72L | 275 | 50.9 |
| 66R | 66696-67106 | 136 | 15.56 | 5.82 |  | 46L | 136 | 99.3 | 63R | 136 | 98.5 | 45L | 136 | 99.3 | 47L | 136 | 98.5 | 71L | 136 | 96.3 |
| 67R | 67234-68106 | 290 | 29.89 | 4.68 |  | 45L | 383 | 61.4 | 64R | 322 | 73.0 | 42L | 85 | 22.1 | 46L | 409 | 58.4 | 70L | 253 | 59.0 |
| 68L^b^ | 68738-72235 | 1165 | 129.19 | 8.34 |  | 44R | 1165 | 98.8 | 65L | 1192 | 96.3 | 41R | 1165 | 98.9 | 45R | 1165 | 98.1 | 69R | 1165 | 97.2 |
| 69L | 72742-73359 | 205 | 22.00 | 9.07 | TM | 43R | 167 | 70.7 | 67L | 227 | 80.2 | 40R | 182 | 75.6 | 43R | 182 | 72.7 | 67R | 207 | 78.3 |
| 70L | 73472-73798 | 108 | 11.84 | 9.57 | Hydrolase of the metallo-beta-lactamase | 42R | 116 | 87.1 | 68L | 116 | 90.5 | 39R | 116 | 87.1 | 42R | 116 | 87.1 | 66R | 88 | 76.9 |
| 71L | 73905-75602 | 565 | 62.19 | 7.91 | Ribonucleoside diphosphate reductase, alpha subunit | 41R | 565 | 98.4 | 69L | 565 | 99.1 | 38R | 565 | 98.2 | 41R | 565 | 98.8 | 65R | 565 | 97.0 |
| 72L^b^ | 75741-76382 | 213 | 23.70 | 10.44 | NIF/NLI interacting factor | 40R | 213 | 97.7 | 70L | 211 | 98.6 | 37R | 209 | 97.2 | 40R | 218 | 95.4 | 64R | 209 | 96.2 |
| 73R | 76777-77067 | 96 | 9.97 | 9.82 |  | 39R | 131 | 37.4 | 71R | 99 | 88.9 | 36L | 207 | 37.2 | 38L | 131 | 38.9 | – |  |  |
| 74R | 77105-77350 | 81 | 9.25 | 8.68 |  | NA |  |  | 72R | 72 | 82.7 | 36L | 207 | 36.2 | – |  |  | 63L | 118 | 39.0 |
| 75L | 77172-77606 | 144 | 14.77 | 7.16 |  | 38R | 91 | 58.3 | NA |  |  | NA |  |  | – |  |  | – |  |  |
| 76R | 77363-77830 | 155 | 15.98 | 10.73 |  | 37R | 153 | 45.8 | 73R | 192 | 59.4 | 35L | 153 | 45.8 | – |  |  | – |  |  |
| 77L | 77917-78237 | 106 | 11.39 | 6.86 | L protein-like protein, TM | 36R | 106 | 98.1 | 74L | 106 | 98.1 | 34R | 106 | 97.2 | NA |  |  | NA |  |  |
| 78L | 78383-78574 | 63 | 6.64 | 8.58 | TM | 35R | 63 | 100.0 | 75L | 63 | 95.2 | 33R | 63 | 98.4 | 34R | 63 | 96.8 | 62R | 63 | 96.8 |
| 79L | 78657-80579 | 640 | 71.10 | 9.98 | Neurofilament triplet H1 protein serine/threonine protein kinase | 34R | 644 | 98.3 | 76L | 716 | 85.1 | 32R | 629 | 89.1 | 33R | 649 | 89.7 | 61R | 738 | 80.6 |
| 80L | 80629-81048 | 139 | 15.17 | 7.84 |  | 33R | 104 | 32.4 | 77L | 139 | 97.1 | 31R | 139 | 97.8 | 32R | 139 | 98.6 | 60R | 139 | 97.8 |
| 81R | 81464-81760 | 98 | 11.29 | 9.84 |  | 31L | 98 | 91.8 | – |  |  | 29L | 98 | 92.9 | 31L | 99 | 73.7 | – |  |  |
| 82L | 81939-82466 | 175 | 19.79 | 8.16 |  | 30R | 162 | 91.4 | 79L | 162 | 91.4 | 28R | 162 | 90.9 | 30R | 162 | 90.3 | 59R | 162 | 87.4 |
| 83L^b^ | 82876-85389 | 837 | 92.47 | 8.09 | Tyrosine kinase/lipopoly-saccride modifying enzyme | 29R | 970 | 79.7 | 80L | 976 | 79.8 | 27R | 970 | 79.5 | 29R | 970 | 79.0 | 58R | 970 | 77.5 |
| 84L | 85948-86649 | 233 | 25.49 | 6.37 | Putative eIF2α-like protein | 28R | 69 | 12.9 | 81L | 270 | 84.4 | 26R | 76 | 16.3 | 27R | 259 | 86.1 | 57R | 259 | 84.6 |
| 85L | 86973-87905 | 310 | 35.26 | 8.43 | p31K protein | 27R | 261 | 83.9 | 82L | 304 | 95.2 | 25R | 262 | 84.2 | 25R | 259 | 82.6 | 55R | 335 | 85.4 |
| 86L | 87953-89050 | 365 | 41.04 | 6.54 |  | 26R | 365 | 97.3 | 83L | 365 | 97.0 | 24R | 365 | 96.4 | 24R | 365 | 92.1 | 54R | 365 | 93.7 |
| 87L | 89436-90584 | 382 | 42.65 | 6.47 |  | 25R | 382 | 98.2 | 84L | 382 | 97.6 | 23R | 382 | 98.4 | 23R | 382 | 96.1 | 53R | 382 | 94.8 |
| 88L^b^ | 90962-93889 | 975 | 108.92 | 6.81 | D5 family NTPase/ATPase | 24R | 975 | 99.4 | 85L | 975 | 99.1 | 22R | 973 | 99.0 | 22R | 975 | 98.6 | 77L | 975 | 98.1 |
| 89R^b^ | 94019-94678 | 219 | 25.44 | 4.70 |  | 23L | 219 | 98.6 | 86R | 222 | 97.3 | 21L | 219 | 100.0 | 21L | 219 | 97.7 | 78R | 219 | 97.7 |
| 90L | 94915-95361 | 148 | 16.00 | 9.24 | TM | 22R | 172 | 82.0 | 87L | 148 | 95.9 | 20R | 148 | 100.0 | 20R | 151 | 92.7 | 79L | 149 | 93.3 |
| 91L^b^ | 95410-98292 | 960 | 104.32 | 9.43 | 2-cysteine adaptor domain | 21R | 852 | 85.4 | 88L | 928 | 93.0 | 19R | 851 | 87.5 | 19R | 931 | 92.7 | 80L | 665 | 65.8 |
| 92R | 98081-98317 | 78 | 8.23 | 5.67 | TM | 20L | 79 | 96.2 | 89R | 78 | 98.7 | 18L | 78 | 96.2 | NA |  |  | NA |  |  |
| 93R | 98354-99862 | 502 | 53.42 | 6.27 |  | 19L | 502 | 99.0 | 90R | 502 | 99.0 | 17L | 502 | 98.8 | 18L | 502 | 98.2 | 81R | 502 | 98.0 |
| 94R | 99899-100846 | 315 | 34.47 | 7.52 |  | 18L | 311 | 97.5 | 92R | 315 | 97.5 | NA |  |  | NA |  |  | 82R | 315 | 95.9 |
| 95L | 100103-100753 | 216 | 23.40 | 10.34 | Integrase-like protein | 17R | 275 | 76.0 | 93L | 275 | 75.6 | 16R | 275 | 76.0 | 17R | 275 | 74.9 | – |  |  |
| 96L^b^ | 101209-102156 | 315 | 35.45 | 5.53 | Putative AAA_ATPase, poxvirus A32 protein family | 16R | 315 | 99.4 | 94L | 315 | 99.0 | 15R | 322 | 97.2 | 16R | 315 | 98.7 | 83L | 308 | 95.6 |
| 97L | 102252-102611 | 119 | 13.44 | 5.08 |  | 15R | 84 | 46.2 | 95L | 119 | 96.6 | 14R | 119 | 96.6 | 15R | 119 | 97.5 | 84L | 124 | 90.3 |
| 98R^b^ | 103378-104271 | 297 | 32.68 | 7.45 |  | 13L | 297 | 98.0 | 98R | 347 | 84.4 | 12L | 297 | 97.3 | 12L | 297 | 98.0 | 87R | 297 | 96.3 |
| 99L | 104337-104549 | 70 | 7.90 | 4.71 | TM | 12R | 70 | 97.1 | 99L | 70 | 100.0 | 11R | 70 | 97.1 | 11R | 70 | 97.1 | 88L | 70 | 94.3 |
| 100R | 105029-105715 | 228 | 24.80 | 5.23 |  | 104R | 223 | 92.5 | 101R | 228 | 98.7 | 96R | 223 | 92.5 | 103R | 223 | 92.5 | 89R | 228 | 94.7 |
| 101R | 105781-106194 | 137 | 15.31 | 5.30 | Putative myeloid cell leukemia protein | 105R | 137 | 97.8 | 102R | 137 | 98.5 | 97R | 137 | 97.8 | 104R | 149 | 88.6 | 90R | 137 | 94.9 |

^a^ TM, transmembrane domain; AA, number of amino acids of each protein; kDa, molecular mass of each protein as predicted by Edit-Seq 5.00; IP, isoelectric points of each protein calculated by Edit-Seq 5.00; ID, identity; NA, the corresponding homologous ORF present but not annotated in the genome; dash (–) mark denotes no corresponding homologous ORF in the genome; ADRV, *Andrias davidianus* ranavirus; RGV, *Rana grylio* virus; CMTV, common midwife toad ranavirus; FV3, frog virus 3; TFV, tiger frog virus; ATV, *Ambystoma tigrinum* virus.

^b^ Core genes of iridoviruses.

^c^ Corresponding homologous ORFs in RGV, CMTV, FV3, TFV, and ATV genomes based on BLASP analysis.

^d^ Amino acid identities were calculated using the Clusta W method in the MegAlign program.
